# Predicting New Zealand riverine fish reference assemblages

**DOI:** 10.7717/peerj.4890

**Published:** 2018-05-28

**Authors:** Adam D. Canning

**Affiliations:** Wellington Fish and Game Council, Palmerston North, New Zealand

**Keywords:** Ecosystem health, Fish barriers, Observed/expected, Nutrients, Riparian, Exotic fish, Biomonitoring, New Zealand, Freshwater, Fish community

## Abstract

Biomonitoring is a common method to monitor environmental change in river ecosystems, a key advantage of biomonitoring over snap-shot physicochemical monitoring is that it provides a more stable, long-term insight into change that is also effects-based. In New Zealand, the main biomonitoring method is a macroinvertebrate sensitivity scoring index, with little established methods available for biomonitoring of fish. This study models the contemporary distribution of common freshwater fish and then uses those models to predict freshwater fish assemblages for each river reach under reference conditions. Comparison of current fish assemblages with those predicted in reference conditions (as observed/expected (O/E) ratios) may provide a suitable option for freshwater fish biomonitoring. Most of the fish communities throughout the central North Island and lower reaches show substantial deviation from the modelled reference community. Most of this deviation is explained by nutrient enrichment, followed by downstream barriers (i.e. dams) and loss of riparian vegetation. The presence of modelled introduced species had relatively little impact on the presence of the modelled native fish. The maps of O/E fish assemblage may provide a rapid way to identify potential restoration sites.

## Introduction

Biomonitoring is the use of biota to detect and track change in an environment and underpins much of the environmental management in developed countries (Friberg et al., 2011; Li, Zheng & Liu, 2010). In aquatic systems, physicochemical monitoring, such as the measurement of nutrient and sediment concentrations, is popular the world over. However, physicochemical monitoring often only provides periodic snapshots of water quality that likely mislead environmental managers on the health of the system (Hazelton, 1998). One common example is where rivers are spot sampled for dissolved oxygen concentration during the day, despite dissolved oxygen having large diurnal fluctuations. Whilst dissolved oxygen may be at sufficient levels during the day, at night (when photosynthesis is not occurring) levels may plummet to stressful or even lethal levels, yet these minima are overlooked by seemingly healthy day-time concentrations (Hazelton, 1998). Whereas biological communities are continually exposed to the ranges of environmental conditions and respond accordingly. Species sensitive to a given disturbance will vacate an area or die, whilst tolerable species will move in or persist, thereby altering the biological community. In New Zealand rivers, nutrient enrichment consistently alters macroinvertebrate communities from one dominated by mayflies, stoneflies and caddisflies to one dominated by chironomid midges, worms and snails (Piggott et al., 2012; Tonkin, Death & Barquín, 2013; Wagenhoff, Townsend & Matthaei, 2012). Biological communities can, therefore, provide a means to detect alterations in environmental conditions without the need for continuous physicochemical monitoring (Friberg et al., 2011; Li, Zheng & Liu, 2010).

In river ecosystems, biomonitoring is most heavily focused at differences in community composition (Friberg et al., 2011). Popular biomonitoring measures of riverine ecosystem health include: taxonomic sensitivity scores, such as Hilsenhoff’s (1988) Family-level Biotic Index in the USA and Stark’s (1993) Macroinvertebrate Community Index (MCI) in New Zealand; metrics that compare species present with those expected to occur under reference conditions (observed/expected (O/E) indicators), such as RIVPACS (Wright, Furse & Armitage, 1993) and RICT (Davy-Bowker et al., 2008) in the UK and the AUSRIVAS (Simpson & Norris, 2000) in Australia; and multi-metric techniques, such as the Index of Biotic Integrity (IBI) applied to periphyton, invertebrate and fish communities throughout many regions around the globe (Karr et al., 1986). Furthermore, in Europe the *European Water Framework Directive* (*2003*) has narratives that assess ecological health using both invertebrates and fish, requiring the management of the composition, abundance and age structure of fish fauna.

The most common river biomonitoring tool in New Zealand is the MCI (Stark, 1993), which indicates on the sensitivity of taxa present to organic enrichment. Other nationally applicable river biomonitoring include periphyton biomass (using chlorophyll *a*) (Biggs & Kilroy, 2000) and the Fish IBI (Joy & Death, 2004). Despite there being regionally applicable predictive biomonitoring (fish O/E indicators) for Taranaki (Joy & Death, 2000) and Manawatu-Wanganui (Joy & Death, 2002), there is still no nationally applicable fish O/E indicator. The latter being a critical missing component in assessing river ecosystem health in New Zealand. A fish O/E for New Zealand would allow environmental managers to not only gauge the number of species lost from a site, but understand what species are likely to be present under reference conditions—this may be useful in assisting setting achievable objectives for river restorations.

Observed/expected indicators rely on the comparison of species present with those expected in reference conditions. At this point, it is important to recognise that reference condition does not necessarily mean human-absent, pre-human or pristine conditions but can be any defined state though often taken to be a close to natural state. Typically, reference communities are predicted to be similar to sites that have been characterised as having similar geomorphology that meet a defined threshold of naturalness, e.g. an upstream native vegetation cover greater than 90%, no exotic species and no human discharges (Friberg et al., 2011; Li, Zheng & Liu, 2010; Wright, Furse & Armitage, 1993). However, it is increasingly difficult to find sites that meet the defined threshold of naturalness across the range of geomorphological make ups, such as lowland streams. Clapcott et al. (2017) show that one way to potentially circumvent the lack of suitable reference sites is to model the communities across all conditions (allowing the use of many sites), allowing for the encapsulation of responses across a gradient of anthropogenic impact. The model can then be used to predict communities at a defined reference state.

Using a similar approach to that by Clapcott et al. (2017), this study aims to develop an O/E indicator for New Zealand riverine fish and decapods. This first involves modelling the current distribution of common fish throughout New Zealand, then predicting fish distribution under defined reference conditions and calculating the O/E ratio for each river reach. A secondary aim is to then explore the potential anthropogenic impacts driving fish O/E scores.

## Methods

### Fish data

Fish and decapod presence absence data were sourced from the New Zealand Freshwater Fish Database (NZFFD) (Richardson, 1989). Only sites that were sampled since 2000 and during the Summer period (December through to March inclusive) using electric fishing over a minimum reach of 150 m (as suggested by Joy, David & Lake (2013)) were included in the study. Restricting our analysis to Summer months is to minimise the influence of migration on predicted distribution. As a result, the predicted probability of fish occurrences only applies to electric fishing in wadable rivers. Furthermore, the influence of temporal changes in fish communities since 2000 were not considered, this represents a trade-off between recent data whilst retaining sufficiently enough surveys of each fish species. Furthermore, Crow et al. (2016) assessed temporal changes in the NZFFD records and found that most species (except Brown trout, Canterbury galaxias and Shortfin Eel) had indeterminate trends between 1977 and 2015. Despite this, Joy (2009, 2014) shows national decline in the Fish IBI (Joy & Death, 2004) at the decadal scale. To ensure a sufficient site selection for the models to learn habitat, only fish species that were present in at least 150 sites were included. Where sites had multiple survey records, only one survey record was included (randomly selected). Overall, 24 native fishes and eight exotic fishes (Table 1) across 19,892 sites were modelled (only native fish were included in the O/E indicator, exotic fish were modelled to explore their impacts).

**Table 1:** All fish species whose distributions were modelled.

Native/exotic	Family	Scientific name	Common name
Native	Anguillidae	*Anguilla australis*	Shortfin eel
		*Anguilla dieffenbachii*	Longfin eel
	Galaxiidae	*Galaxias argenteus*	Giant kokopu
		*Galaxias brevipinnis*	Koaro
		*Galaxias divergens*	Dwarf galaxias
		*Galaxias fasciatus*	Banded kokopu
		*Galaxias maculatus*	Inanga
		*Galaxias postvectis*	Shortjaw kokopu
		*Galaxias anomalus*	Roundhead galaxias
		*Galaxias depressiceps*	Flathead galaxias
		*Galaxias gollumoides*	Gollum galaxias
		*Galaxias vulgaris*	Canterbury galaxias
		*Galaxias paucispondylus*	Alpine galaxias
	Geotriidae	*Geotria australis*	Lamprey
	Eleotridae	*Gobiomorphus basalis*	Crans bully
		*Gobiomorphus breviceps*	Upland bully
		*Gobiomorphus gobioides*	Giant bully
		*Gobiomorphus hubbsi*	Bluegill bully
		*Gobiomorphus huttoni*	Redfin bully
		*Gobiomorphus cotidianus*	Common bully
	Pinguipedidae	*Cheimarrichthys fosteri*	Torrent fish
	Decapoda	*Paranephrops spp.*	Koura
		*Paratya curvirostris*	Freshwater shrimp
	Pleuronectidae	*Rhombosolea retiaria*	Black flounder
Exotic	Salmonidae	*Oncorhynchus tshawytscha*	Chinook salmon
		*Oncorhynchus mykiss*	Rainbow trout
		*Salmo trutta*	Brown trout
	Percidae	*Perca fluviatilis*	Perch
	Cyprinidae	*Carassius auratus*	Goldfish
		*Scardinius erythrophthalmus*	Rudd
	Ictaluridae	*Ameiurus nebulosus*	Catfish
	Poeciliidae	*Gambusia affinis*	Gambusia

**Note:**

All natives were included in the observed/expected indicator, whilst exotics were included in the impact assessment.

### Environmental data

At each river reach, most environmental variables were extracted from the Freshwater Environments New Zealand (FENZ) geodatabase (Leathwick et al., 2010), except for the nitrate-nitrogen (N) and dissolved reactive phosphorus (DRP) predicted concentrations which were sourced from Unwin & Larned (2013), and the hydrological characteristics which were sourced from Booker & Woods (2014) (Table 2).

**Table 2:** The environmental variables used to model fish distributions with the mean and range of values across the fish dataset.

Characteristic group	Metric	Definition	Mean	Min	Max
Land cover	USLake	Proportion of upstream catchment covered by lake	0.00	0.00	0.69
	USNative	Proportion of upstream catchment covered by native vegetation	0.54	0.00	1.00
	USPasture	Proportion of upstream catchment covered by pasture	0.35	0.00	1.00
	USPeat	Proportion of upstream catchment covered by peat	0.01	0.00	1.00
	USLake	Proportion of upstream catchment covered by lake	0.01	0.00	1.00
	USGlacier	Proportion of upstream catchment covered by glacier	0.00	0.00	0.85
	SegRipNative	Proportion of native riparian vegetation within a 100 m buffer of the river	40.29	0.00	100.10
Catchment geology	USHardness	Average hardness of rocks in the catchment, 1 = very low to 5 = very high	3.10	0.00	5.00
	USCalcium	Average calcium concentration of rocks in the catchment, 1 = very low to 4 = very high	1.49	0.00	4.00
	USPhosporus	Average phosphorus concentration of rocks in the catchment, 1 = very low to 5 = very high	2.47	0.00	5.00
Climate	SegRipShade	The likely proportion of stream shaded from riparian	0.42	0.00	0.80
	SegJanAirT	Summer (January) air temperature (°C)	16.08	0.00	19.80
	SegMinTNorm	Average minimum daily air temperature (°C) normalised with respect to SegJanAirT	0.26	−4.26	26.83
	USAvgTNorm	Average air temperature (°C) in the upstream catchment, normalised with respect to SegJanAirT	−0.20	−7.85	135.40
	USDaysRain	Days per year with rainfall greater than 25 mm in the upstream catchment	15.36	1.20	104.60
River characteristics	ReachHab	Weighted average of proportional cover of local habitat using categories of: 1 = still; 2 = backwater; 3 = pool; 4 = run; 5 = riffle; 6 = rapid; 7 = cascade	3.97	1.10	6.10
	ReachSed	Weighted average of proportional cover of bed sediment using categories of: 1 = mud; 2 = sand; 3 = fine gravel; 4 = coarse gravel; 5 = cobble; 6 = boulder; 7 = bedrock	3.60	0.00	6.50
	SegSlope	Slope of segment (°)	1.94	0.00	29.70
	USAvgSlope	Average slope (°) in the upstream catchment	13.63	0.00	44.33
	DSAvgSlope	Average slope (°) in the downstream catchment	0.53	0.00	51.42
	DSDam	The presence (1) or absence (0) of downstream obstructions (mainly dams)	0.20	0.00	1.00
	DSMaxLocalSlope	Maximum downstream slope (degrees), local slopes at 100 m intervals along each river segment were calculated and maximum value encountered recorded	8.06	0.00	54.11
	DSDist2Coast	Distance to coast (km) from mid-point of each river segment	79.51	0.01	432.84
	NO3N_State	Predicted nitrate-nitrogen concentration	0.25	0.00	5.54
	DRP_State	Predicted dissolved reactive phosphorus concentration	0.01	0.00	0.10
Hydrological characteristics	Feb	Mean daily February flow divided by the overall mean daily flow	0.60	0.24	1.62
	FRE3	Predicted annual frequency of flows exceeding three times the annual median flow	14.51	1.81	38.98
	MALF	The seven day mean annual low flow (cumecs)	2.04	0.00	442.82
	MeanF	Mean of all daily flows (cumecs)	6.81	0.00	1327.73
	Q5	One in five year seven-day mean annual low flow (cumecs)	1.60	0.00	317.91
	WidthMALF	Predicted wetted width (m) at MALF	4.98	0.06	117.80
	WidthQ5	Predicted wetted width (m) at Q5	4.67	0.05	110.97

The FENZ geodatabase also classifies river reaches into groups with similar environmental conditions (Leathwick et al., 2010, 2011). The classifications were made using Generalised Dissimilarity Modelling that used the FENZ geodatabase to explain the biological dissimilarity in fish and macroinvertebrate distributions (Leathwick et al., 2011). To reduce the risk of over-extrapolation, only river classes that contained at least 1,000 fish survey sites were included in the exercise, thus all predictions are restricted only to those river classes (A, C, G and H) where sufficient records exist.

### Fish distribution models

Using all records from FENZ river classes with at least 1,000 survey sites, each of the 24 native fish and decapod species and eight exotic species included in the study (Table 1) were modelled from all environmental variables (Table 2) using boosted regression tree (BRT) models. BRT models consist of many simple tree models that when combined can fit complex relationships (Elith, Leathwick & Hastie, 2008). BRT models are capable of fitting interactions and non-linear predictors, can handle non-normal error terms and missing values, and can identify the most informative predictors whilst ignoring irrelevant ones (Elith, Leathwick & Hastie, 2008; Friedman & Meulman, 2003). Tree complexity was set at five, whilst the learning rate was set to ensure that at least 1,000 trees were assembled, as recommended by Elith, Leathwick & Hastie (2008). Models were cross-validated with a bag fraction of 0.15 and 10 K-folds. Model performance was assessed using the cross-validated area under the receiver operator curve (AUC). Linear regressions were used to screen co-linearity between predictor variables (Table S1).

The BRT models also give the relative importance of environmental variables for each species. Using the relative influence of the five most important variables for each species, non-metric multidimensional scaling (NMDS) was used to portray the dissimilarity (Euclidean method) in the important environmental variables predicting each species. The NMDS was produced using the PAST3 software package (Hammer, Harper & Ryan, 2001).

To estimate current fish distribution, each of the BRT models were extrapolated across all New Zealand river reaches within the FENZ classes (A, C, G and H). Following a similar approach to Clapcott et al. (2017), fish distribution at each reach in reference conditions was estimated by setting: the proportion of upstream and riparian native cover were set to 100%; the proportion of upstream pasture set to 0%; the proportion of riparian shade was set to that estimated assuming complete vegetation in pre-human conditions as published in FENZ; the predicted N and DRP concentrations were reduced (where exceeded) to 0.11 and 0.006 mg/L respectively, as these reflect the upper range of nutrient enrichment for river reaches with high ecological health as defined by Death et al. (2018).

The BRT models predict the probability of species capture for each reach. Many models assume that species are present when the probability of capture is greater than 0.5; however, unless the data used to create the model is balanced (i.e. species occurrence at ∼50% of sites) then a threshold of 0.5 would not provide the best representation. To circumvent this, Schroder (2004) was used to compare actual species presence–absence with predicted probability of capture and calculate the Cohen’s Kappa for all potential thresholds between zero and one in 0.005 steps. The threshold that maximised Cohen’s Kappa for a given species was selected as the threshold with the best prediction. Fish were considered present at a site when their probability of capture was equal to or exceeded the threshold selected for that species.

**Figure 1:**
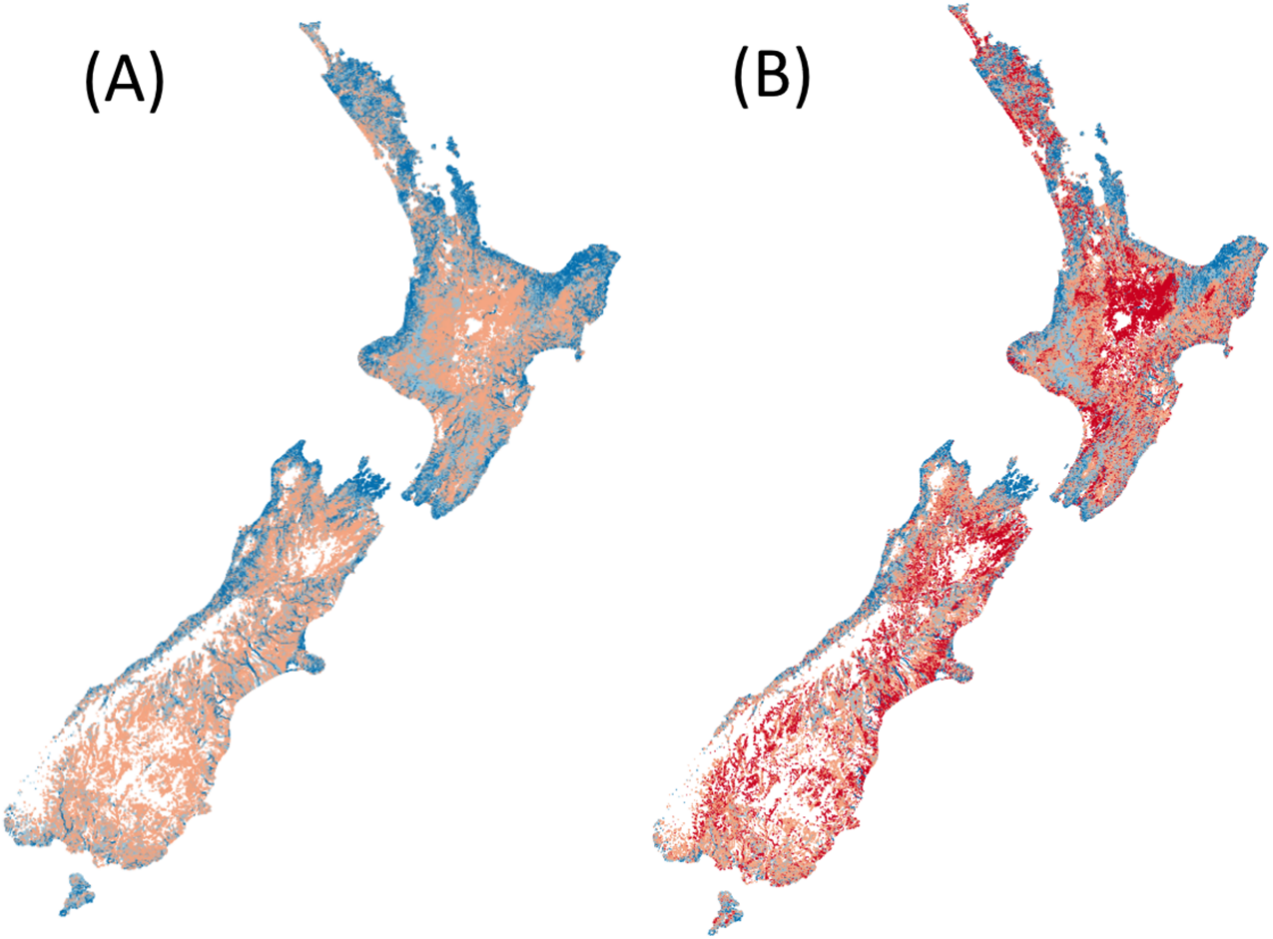
Predicted species richness of fish in both (A) reference conditions and (B) present conditions. High species richness represented by dark blue, moderate richness by orange and low or no species by red.

**Figure 2:**
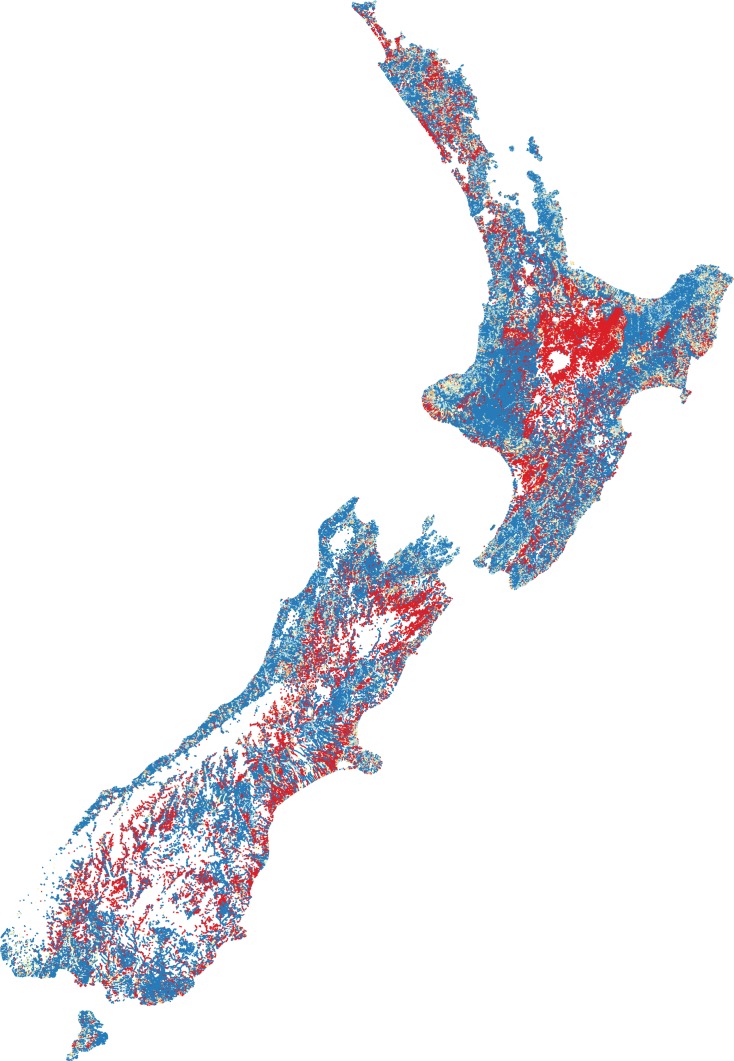
Predicted fish observed/expected ratio throughout New Zealand. Ratios close to one are represented by blue through to ratios close to zero represented by red. Orange and yellow hues represent moderate ratios.

The O/E ratio was predicted for each river reach by:

1. Counting the number of native fish species (from the 24 modelled) that are predicted (i.e. expected) to occur in reference conditions (Fig. 1A).
2. Counting the number of native fish species (from the 24 modelled) that are predicted to be present in present-day conditions and were predicted to occur in human-absent conditions (i.e. observed) (Fig. 1B).
3. Dividing the number of observed species by the number of species expected gives a ratio between zero and one (Fig. 2). High ratios indicate that fish presence assemblages are similar to those expect in reference conditions, whilst low ratios suggest fish presence assemblages are substantially different from human-absent conditions.

### Comparison with Crow et al. (2014)

Crow et al. (2014) used Regularized Random Forest models to predict the present-day New Zealand-wide distribution of freshwater fishes, also using data from the NZFFD. As a comparison, the percent agreement (of fish presence–absence) between the predicted current fish distributions (i.e. the observed) derived in this study and those developed by Crow et al. (2014) were ascertained for all river reaches for 28 species.

### Human impacts on the predicted fish observed/expected ratio

The fish O/E was predicted for 208,449 FENZ river reaches. The predicted O/E for each river was modelled using the same BRT procedure described above, but predicted from the following human-influenced factors: The presence/absence of a downstream dam (Leathwick et al., 2010); predicted nitrate-nitrogen and DRP concentrations (Unwin & Larned, 2013); predicted *E. Coli* concentrations (Unwin & Larned, 2013); the O/E riparian vegetation cover (Leathwick et al., 2010); the O/E fine sediment cover (Clapcott et al., 2011); and the predicted presence of exotic *Oncorhynchus mykiss* (Rainbow Trout), *Salmo trutta* (Brown Trout), *Perca fluviatilis* (Perch), *Scardinius erythrophthalmus* (Rudd), *Carassius auratus* (Goldfish), *Gambusia affinis* (Gambusia), *Oncorhynchus tshawytscha* (Chinook salmon) and *Ameiurus nebulosus* (Catfish).

## Results

**Figure 3:**
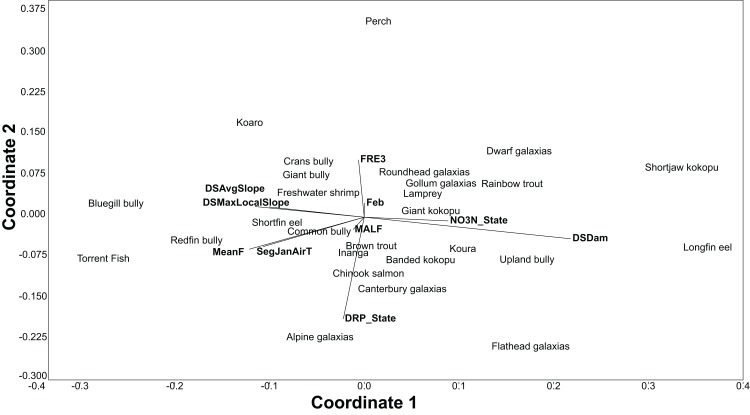
An NMDS of the dissimilarity (Euclidian) in influential environmental variables for predicting freshwater fish distribution, as determined by the BRT models.

All fish modelled had good or excellent performances as measured by the AUC (>0.8 and >0.9 respectively; Table 3). The best thresholds for species presence range from 0.025 to 0.48. Slope, air temperature, nutrients and flood frequency were among the most common influential factors determining fish distribution (Table 3; Fig. 3).

**Table 3:** Boosted Regression Tree distribution models of 32 riverine taxa throughout New Zealand’s North Island.

Common name	Most influential factors					Relative contribution (%)						
	Factor 1	Factor 2	Factor 3	Factor 4	Factor 5	AUC	Cont. 1	Cont. 2	Cont. 3	Cont. 4	Cont. 5	Agreement (%)
Shortfin eel	SegJanAirT	DSMaxLocalSlope	NO3N_State	SegMinTNorm	DRP_State	0.86	40.1	11.0	8.5	3.5	2.9	86
Longfin eel	USNative	SegJanAirT	SegMinTNorm	NO3N_State	DSDam	0.82	14.1	9.4	7.3	6.1	5.3	71
Giant kokopu	FRE3	SegMinTNorm	DSMaxLocalSlope	USDaysRain	SegJanAirT	0.90	22.6	10.8	9.7	4.8	4.6	99
Koaro	DSAvgSlope	USAvgSlope	FRE3	SegJanAirT	USDaysRain	0.88	10.3	7.1	6.9	6.7	6.2	89
Dwarf galaxias	SegMinTNorm	DSMaxLocalSlope	SegJanAirT	USDaysRain	USAvgSlope	0.96	13.1	10.8	7.6	6.8	6.2	98
Alpine galaxias	DRP_State	USCalcium	SegJanAirT	SegMinTNorm	DSMaxLocalSlope	0.98	24.5	13.6	7.1	5.8	4.2	99
Flathead galaxias	DSMaxLocalSlope	USDaysRain	SegJanAirT	USPhosporus	SegMinTNorm	0.99	13.4	11.3	10.2	8.3	7.3	100
Canterbury galaxias	DRP_State	FRE3	SegMinTNorm	DSAvgSlope	USCalcium	0.97	13.6	12.3	9.5	9.3	8.6	97
Gollum galaxias	SegJanAirT	NO3N_State	SegMinTNorm	USDaysRain	DSMaxLocalSlope	0.99	10.7	10.1	8.5	8.4	7.4	99
Banded kokopu	DSAvgSlope	SegMinTNorm	SegJanAirT	SegRipShade	FRE3	0.92	22.7	9.3	8.3	7.3	6.3	90
Inanga	DSMaxLocalSlope	SegJanAirT	DSAvgSlope	SegMinTNorm	FRE3	0.89	28.5	11.1	7.5	6.7	4.2	90
Shortjaw kokopu	FRE3	USNative	SegMinTNorm	USDaysRain	USPhosporus	0.93	9.8	9.3	6.7	6.2	6.1	99
Roundhead galaxias	DSMaxLocalSlope	FRE3	USDaysRain	SegMinTNorm	Feb	0.99	21.0	11.1	10.9	7.9	5.7	100
Lamprey	DSMaxLocalSlope	DSAvgSlope	SegMinTNorm	USDaysRain	DRP_State	0.85	8.9	5.8	5.8	5.4	5.2	99
Crans bully	SegJanAirT	DSAvgSlope	Feb	DSMaxLocalSlope	USDaysRain	0.94	13.9	9.5	9.3	8.9	5.8	89
Upland bully	SegMinTNorm	SegJanAirT	DSMaxLocalSlope	FRE3	USPhosporus	0.93	16.4	13.0	10.9	6.7	5.6	90
Common bully	DSMaxLocalSlope	SegJanAirT	FRE3	SegMinTNorm	MeanF	0.83	19.4	10.1	5.1	4.9	4.1	95
Giant bully	DSMaxLocalSlope	SegJanAirT	DSAvgSlope	FRE3	USDaysRain	0.91	14.3	12.7	10.5	8.4	5.0	99
Bluegill bully	DSMaxLocalSlope	FRE3	USAvgTNorm	USAvgSlope	SegJanAirT	0.93	8.3	8.2	7.1	6.4	6.3	95
Redfin bully	FRE3	DSMaxLocalSlope	USAvgTNorm	SegJanAirT	DSAvgSlope	0.92	9.3	8.1	6.7	6.6	6.2	77
Torrent Fish	USAvgTNorm	SegJanAirT	DSMaxLocalSlope	MeanF	DSAvgSlope	0.90	13.1	11.7	9.9	7.6	6.0	93
Koura	DRP_State	FRE3	SegMinTNorm	Feb	USPhosporus	0.85	11.5	9.0	8.7	7.1	6.6	N/A
Freshwater shrimp	SegJanAirT	FRE3	DSMaxLocalSlope	USDaysRain	SegMinTNorm	0.90	24.5	9.0	7.3	5.4	5.1	N/A
Chinook salmon	DRP_State	DSMaxLocalSlope	SegMinTNorm	USAvgTNorm	NO3N_State	0.88	14.9	11.5	9.5	6.1	4.7	99
Rainbow trout	FRE3	DSAvgSlope	SegMinTNorm	USDaysRain	USPhosporus	0.91	10.2	9.5	8.8	7.5	6.8	95
Brown trout	SegJanAirT	MeanF	DSAvgSlope	SegMinTNorm	MALF	0.86	19.8	8.1	6.2	5.8	5.3	95
Perch	USDaysRain	DSMaxLocalSlope	FRE3	USLake	USPhosporus	0.91	9.0	6.4	6.3	5.9	5.7	78
Black flounder	DSMaxLocalSlope	Feb	FRE3	Q5	MeanF	0.92	15.9	6.4	6.0	5.9	5.6	100
Rudd	USLake	DSAvgSlope	USPhosporus	Feb	USNative	0.95	16.0	13.9	7.7	7.6	5.3	N/A
Goldfish	DSAvgSlope	USLake	SegJanAirT	DRP_State	USPeat	0.94	13.0	10.7	7.9	7.1	5.1	99
Catfish	DSAvgSlope	USPhosporus	USPeat	USLake	USWetland	0.94	12.7	9.5	8.1	6.8	5.9	N/A
Gambusia	SegJanAirT	USAvgSlope	ReachSed	Feb	DRP_State	0.96	23.0	15.8	4.5	4.3	3.9	98

When the predicted current fish distributions were compared with those derived by Crow et al. (2014), on average there was 93% agreement in the predicted presence–absence of a species at a reach. The percentage agreement for individual species ranged from 71% to 100% and are shown in Table 3.

The O/E was, perhaps not surprisingly, greatest in naturally forested areas followed by areas dominated by low intensity agriculture. The lowest scores were in primarily in the central North Island (Waikato and Manawatu regions) where intensive agriculture and (in the case of Waikato) many dams (primarily for hydropower generation and irrigation) exist. BRT modelling of the predicted O/E values had a cross-validated correlation coefficient of 0.56 and suggests that DRP concentration (26.4%), NO3-N concentration (21.6%), downstream dams (20.7%), O/E riparian cover (16.1%) and O/E sediment cover (8.0%) are among the most influential factors in predicting the O/E, with exotic fish, such as Rainbow Trout (1%), Goldfish (0.5%), Perch (0.03%) and Brown Trout (0.005%), having relatively little influence (as indicated by percentages).

## Discussion

The BRT modelling successfully predicted the distribution of all 32 species (native and exotic) with high accuracy. Geographic and climatic variables were the primary drivers of New Zealand freshwater fish distribution, this is consistent with the findings of Joy & Death (2002) and Leathwick et al. (2005). Furthermore, the BRT models were optimised using cross-validation, which has been shown to have substantially less bias than re-substitution approaches or simple training/test splits (Olden & Jackson, 2002; Olden, Jackson & Peres-Neto, 2002); the predictions also had high inter-rater agreement with the predictions by Crow et al. (2014); thus, instilling confidence in the predictions.

Whilst the models performed well in predicting each species, each species was modelled independently of each other. This means that there is spatial autocorrelation between individual species and model parameters but not with other native species that typically co-occur. Other approaches that model whole communities have found that they may provide greater predictive accuracy, particularly for rare species, by learning when species co-exist (Nieto-Lugilde et al., 2018; Olden, Joym & Death, 2006), this may be an interesting comparison for future analysis.

The vast majority of New Zealand lowland streams have some degree of human impact. This may have led the BRT models to associate natural characteristics of lowland streams, such as distance to coast and slope, with fish distributions that are actually the result of human impacts. However, given that lowland streams differ substantially in their degree of human impact, it is also plausible that the gradient of impact on fish distribution is well encapsulated, resulting in reasonable predictions under more natural conditions. The predicted reference conditions do not represent natural conditions but provide a benchmark that is close to natural conditions. Another key issue is that the defined reference condition could not account for changes in geomorphology (physical habitat), primarily due to a lack of data on geomorphological reference condition. This may be somewhat alleviated by physical habitat likely correlating with land use, which was defined in the reference condition. Nevertheless, not all changes in physical habitat are the result of land use, with flood management engineering being another key driver of physical habitat change. The river environment classification (REC) Geographic information system (GIS) layers used are also representative of current river extent, the definition of reference condition did not account for the straightening of rivers, as such the extent of river reaches in reference condition is defined by the REC, rather than historical/pre-human extent. Despite these pitfalls, Clapcott et al. (2017) showed that an almost identical approach to that recruited here was accurate in predicting the MCI in close to natural conditions. Still, this risk is one of the unfortunate disadvantages of having very little lowland sites in natural condition. Future of comparison of this approach with traditional O/E approaches (using representative sites in reference condition) would also be worthwhile to observe the influence of reference definition on O/E outcomes.

The BRT models are also prone to data uncertainty from several sources. First, the success of electric fishing can be heavily impacted by user skill, water conductivity and machine settings. The freshwater fish database does not include data on these aspects to allow for their control. However, these impacts were minimised by only selecting reaches that had at least 150 m surveyed, as recommended by Joy, David & Lake (2013), and only assessing fish presence/absence rather than relative abundance or density. Second, many New Zealand freshwater fish are migratory and may be absent from streams at various times of year (McDowall, 1990); also, many fish become less active at colder temperatures making them harder to catch (David & Closs, 2003; Jellyman, 1991; Jellyman, Glova & Todd, 1996; Ryan, 1984). Temporal variability can also present from sampling that occurred soon after a flood (David & Closs, 2002). Whilst it was not possible for surveys following recent floods to be identified and removed, seasonal impacts were reduced by excluding data collected from May to October (inclusive) to maximise species presence (Joy, David & Lake, 2013).

The predicted O/E suggests the vast majority of absences are throughout the central North Island (predominantly the Waikato and Manawatu regions), followed by lowland flat areas. The presence of downstream dams and heightened nutrient enrichment were the most influential anthropogenic factors impacting the O/E, followed by riparian loss and sedimentation. Interestingly, introduced salmonids and perch had relatively very little influence on the O/E of native fish. Due to data limitations, this study could not assess the loss of physical habitat on fish O/E; the loss of physical habitat is likely to be a large driver of fish exclusion and warrants further exploration.

Many New Zealand fish are diadromous (Joy & Death, 2001; McDowall, 1998a, 1998b) and it is probable that the inhibition of migration is the key factor underlying the high influence of downstream dams. Baker (2003) found that juvenile migratory galaxiids and common bullies were restricted by weir fall heights as little as 10 cm, whilst adult galaxiids were restricted by falls greater than 20 cm. Furthermore, Joy & Death (2001) examined 85 sites across 38 streams with dams or weirs in the Taranaki region and found that fish species richness was consistently higher downstream than upstream of dams. The predictions of expected (reference condition) fish distribution could be used to prioritise fish passage improvement at dams where there is a large area of upstream catchment potentially habitable to multiple species.

New Zealand freshwater ecosystems are primarily threatened by agricultural intensification, which has driven large increases in excess nitrogen, phosphorus and sediment. Excess nitrogen and phosphorus can impact fish community health either directly at physiologically toxic levels (e.g. nitrite toxicity) or, more commonly, indirectly by permitting excessive algal growth. High algal growth often alters macroinvertebrate communities from one dominated by mayflies, caddisflies and stoneflies to one dominated by chironomids midges, snails and worms—the latter community being less energetically rewarding for fish. The extra algal growth and decomposition can also increase diurnal oxygen fluctuations resulting in stressful hyperoxic and hypoxic conditions. Though, excessive growth can be mediated by riparian shading.

Riparian vegetation can benefit fish communities by reducing algal growth, sustaining allochthonous inputs and supporting diverse habitat. Riparian vegetation can intercept and absorb nutrients flowing into the river as well as shade the river bed—both can limit the growth and consequences of excessive algal growth. High leaf litter and terrestrial invertebrate inputs from vegetated riparian can also sustain shredding invertebrates (which fish can then consume) and support the diets of some fish species. For example, Bonnett & Lambert (2002) found that terrestrial invertebrates occurred in 83% of Giant Kokopu (*Galaxias argenteus*) stomachs, and comprised 25% of the gut content. Riparian vegetation can also create and maintain habitat diversity by stabilising banks, shading streams, regulating temperature and providing root structures and woody debris (Pusey & Arthington, 2003).

Interestingly, the presence of the introduced species, including the very widespread Brown Trout, had almost negligible influence on the O/E of native fish. Previous studies have suggested that salmonids have replaced non-migratory galaxiids in some streams (but not others) and reduce the relative abundance of large and drifting invertebrates (McIntosh & Townsend, 1996; McIntosh, Townsend & Crowl, 1992; Townsend, 2003). However, this study did not include all non-migratory galaxiids as they have restricted distributions with too few surveys to be modelled. Furthermore, this study only assessed the presence or absence of species, rather than changes in abundance. Whilst the presence of exotic fish may not have marked impacts on the presence of species assessed, brown trout have been associated with reduced galaxiid abundance and size (McIntosh, Crowl & Townsend, 1994; McIntosh et al., 2010; Olsson et al., 2006). Therefore, an O/E of species abundance rather than presence–absence may be more sensitive to the impacts of introduced species.

## Conclusions

In conclusion, this study presents a presence–absence fish O/E indicator that is applicable for the majority of New Zealand river reaches. It may be useful for the rapid assessment of native fish communities and may be useful for identifying potential restoration sites. It is shown that barriers, such as dams, and nutrient enrichment have considerable influence over the distribution of native fish. Further work is needed to assess the impact of physical habitat change on the exclusion of native fish.

## Supplemental Information

10.7717/peerj.4890/supp-1 Supplemental Information 1 Table S1. Pearson correlations (top) and p-values (bottom) for regressions between each of the predictors used to model fish distribution.

## Additional Information and Declarations

### Data Availability

The following information was supplied regarding data availability: Canning, Adam (2018): All_Spp_PA_OE_FENZ_classes.csv.figshare. Dataset. https://doi.org/10.6084/m9.figshare.5830932.v1.
