# Supplementary material for: Predicting New Zealand riverine fish reference assemblages
Source: PeerJ. 2018 May 28;6:e4890. doi: 10.7717/peerj.4890 (PMC5978389; doi:10.7717/peerj.4890)
Supplement: Supplemental Information 1 [file peerj-06-4890-s001.docx]

| **Table S1.** Pearson correlations (top) and p-values (bottom) for regressions between each of the predictors used to model fish distribution. | | | | | | | | | | | | | | | | | | | | | | | | | | | | | | | | |
| --- | --- | --- | --- | --- | --- | --- | --- | --- | --- | --- | --- | --- | --- | --- | --- | --- | --- | --- | --- | --- | --- | --- | --- | --- | --- | --- | --- | --- | --- | --- | --- | --- |
|  | SegJanAirT | SegMinTNorm | SegSlope | SegRipShade | SegRipNative | DSAvgSlope | DSMaxLocalSlope | DSDam | USAvgTNorm | USDaysRain | USAvgSlope | USCalcium | USHardness | USPhosporus | USPeat | USLake | USWetland | USNative | USPasture | USGlacier | ReachSed | ReachHab | DRP_State | NO3N_State | Q5 | MALF | MeanF | Feb | WidthQ5 | WidthMALF | FRE3 |  |
| SegJanAirT |  | 0.18 | -0.13 | 0.07 | -0.28 | 0.04 | -0.36 | -0.24 | -0.30 | -0.01 | -0.20 | -0.03 | -0.26 | -0.39 | 0.06 | 0.04 | -0.02 | -0.34 | 0.23 | -0.07 | -0.38 | -0.26 | 0.38 | 0.20 | 0.01 | 0.01 | 0.01 | -0.33 | 0.02 | 0.02 | 0.06 |  |
| SegMinTNorm | 0 |  | -0.10 | 0.16 | -0.04 | 0.18 | -0.33 | -0.56 | 0.31 | 0.30 | -0.20 | -0.14 | -0.19 | -0.23 | 0.11 | 0.00 | 0.05 | -0.16 | 0.10 | -0.09 | -0.13 | -0.19 | 0.28 | 0.13 | -0.03 | -0.03 | -0.03 | -0.05 | -0.08 | -0.08 | 0.48 |  |
| SegSlope | 0 | 0 |  | 0.31 | 0.35 | 0.25 | 0.50 | 0.10 | -0.15 | 0.09 | 0.34 | -0.05 | 0.29 | 0.05 | -0.07 | -0.05 | -0.05 | 0.28 | -0.25 | -0.03 | 0.36 | 0.34 | -0.24 | -0.23 | -0.06 | -0.06 | -0.06 | 0.14 | -0.16 | -0.16 | 0.07 |  |
| SegRipShade | 0 | 0 | 0 |  | 0.43 | 0.20 | 0.15 | -0.08 | 0.29 | 0.04 | -0.06 | -0.07 | 0.05 | -0.15 | -0.02 | -0.12 | 0.01 | 0.09 | -0.22 | -0.14 | -0.05 | -0.12 | 0.00 | -0.14 | -0.17 | -0.17 | -0.17 | -0.09 | -0.40 | -0.41 | 0.19 |  |
| SegRipNative | 0 | 0 | 0 | 0 |  | 0.15 | 0.39 | 0.13 | -0.03 | 0.21 | 0.37 | -0.23 | 0.33 | -0.03 | -0.03 | -0.02 | 0.07 | 0.71 | -0.61 | -0.04 | 0.41 | 0.31 | -0.39 | -0.44 | -0.08 | -0.08 | -0.08 | 0.19 | -0.11 | -0.11 | 0.18 |  |
| DSAvgSlope | 0 | 0 | 0 | 0 | 0 |  | 0.11 | -0.09 | 0.02 | 0.11 | 0.09 | -0.10 | 0.13 | -0.07 | -0.03 | -0.01 | -0.03 | 0.10 | -0.10 | -0.02 | 0.10 | 0.04 | -0.05 | -0.08 | -0.03 | -0.03 | -0.03 | 0.02 | -0.07 | -0.08 | 0.11 |  |
| DSMaxLocalSlope | 0 | 0 | 0 | 0 | 0 | 0 |  | 0.44 | -0.09 | 0.02 | 0.35 | -0.05 | 0.31 | 0.06 | -0.12 | 0.00 | -0.07 | 0.37 | -0.33 | 0.02 | 0.42 | 0.39 | -0.31 | -0.35 | -0.06 | -0.05 | -0.06 | 0.19 | -0.07 | -0.07 | -0.12 |  |
| DSDam | 0 | 0 | 0 | 0 | 0 | 0 | 0 |  | -0.11 | -0.12 | 0.10 | 0.06 | 0.11 | 0.16 | -0.05 | 0.05 | 0.00 | 0.20 | -0.17 | 0.10 | 0.10 | 0.13 | -0.16 | -0.16 | -0.01 | -0.01 | -0.01 | 0.12 | 0.01 | 0.01 | -0.24 |  |
| USAvgTNorm | 0 | 0 | 0 | 0 | 0 | 0.017 | 0 | 0 |  | -0.16 | -0.51 | 0.12 | -0.25 | 0.02 | 0.08 | -0.06 | 0.07 | -0.29 | 0.25 | -0.27 | -0.32 | -0.36 | 0.30 | 0.24 | -0.30 | -0.29 | -0.30 | -0.42 | -0.56 | -0.56 | 0.08 |  |
| USDaysRain | 0.465 | 0 | 0 | 0 | 0 | 0 | 0.029 | 0 | 0 |  | 0.26 | -0.22 | -0.03 | -0.07 | 0.00 | 0.04 | 0.04 | 0.33 | -0.31 | 0.18 | 0.24 | 0.24 | -0.17 | -0.17 | 0.03 | 0.03 | 0.04 | 0.44 | 0.15 | 0.15 | 0.81 |  |
| USAvgSlope | 0 | 0 | 0 | 0 | 0 | 0 | 0 | 0 | 0 | 0 |  | -0.29 | 0.60 | -0.05 | -0.17 | -0.02 | -0.13 | 0.64 | -0.57 | 0.18 | 0.61 | 0.64 | -0.61 | -0.56 | 0.11 | 0.11 | 0.13 | 0.43 | 0.24 | 0.24 | 0.05 |  |
| USCalcium | 0 | 0 | 0 | 0 | 0 | 0 | 0 | 0 | 0 | 0 | 0 |  | -0.20 | 0.34 | 0.06 | -0.08 | 0.01 | -0.33 | 0.38 | -0.11 | -0.13 | -0.13 | 0.26 | 0.30 | -0.02 | -0.02 | -0.01 | -0.18 | -0.08 | -0.07 | -0.22 |  |
| USHardness | 0 | 0 | 0 | 0 | 0 | 0 | 0 | 0 | 0 | 0 | 0 | 0 |  | 0.02 | -0.21 | -0.03 | -0.14 | 0.46 | -0.42 | 0.06 | 0.49 | 0.45 | -0.59 | -0.41 | 0.02 | 0.02 | 0.03 | 0.14 | 0.05 | 0.06 | -0.07 |  |
| USPhosporus | 0 | 0 | 0 | 0 | 0 | 0 | 0 | 0 | 0.014 | 0 | 0 | 0 | 0.004 |  | -0.13 | -0.07 | -0.02 | 0.04 | 0.09 | 0.01 | 0.16 | 0.13 | -0.18 | 0.14 | 0.00 | 0.00 | 0.00 | 0.12 | -0.02 | -0.02 | -0.13 |  |
| USPeat | 0 | 0 | 0 | 0.005 | 0 | 0 | 0 | 0 | 0 | 0.831 | 0 | 0 | 0 | 0 |  | 0.02 | 0.33 | -0.08 | 0.09 | -0.01 | -0.23 | -0.23 | 0.26 | 0.08 | -0.01 | -0.01 | -0.01 | -0.03 | -0.03 | -0.03 | 0.05 |  |
| USLake | 0 | 0.631 | 0 | 0 | 0.001 | 0.326 | 0.566 | 0 | 0 | 0 | 0.004 | 0 | 0 | 0 | 0.008 |  | 0.03 | -0.01 | -0.04 | 0.08 | -0.06 | -0.06 | -0.03 | -0.05 | 0.22 | 0.22 | 0.21 | 0.08 | 0.26 | 0.26 | -0.02 |  |
| USWetland | 0.016 | 0 | 0 | 0.426 | 0 | 0 | 0 | 0.544 | 0 | 0 | 0 | 0.157 | 0 | 0.01 | 0 | 0 |  | 0.05 | -0.04 | -0.01 | -0.12 | -0.13 | 0.07 | -0.03 | -0.01 | -0.01 | -0.01 | 0.00 | -0.02 | -0.02 | 0.08 |  |
| USNative | 0 | 0 | 0 | 0 | 0 | 0 | 0 | 0 | 0 | 0 | 0 | 0 | 0 | 0 | 0 | 0.159 | 0 |  | -0.83 | 0.10 | 0.58 | 0.49 | -0.63 | -0.62 | 0.04 | 0.04 | 0.04 | 0.38 | 0.12 | 0.11 | 0.23 |  |
| USPasture | 0 | 0 | 0 | 0 | 0 | 0 | 0 | 0 | 0 | 0 | 0 | 0 | 0 | 0 | 0 | 0 | 0 | 0 |  | -0.09 | -0.51 | -0.41 | 0.57 | 0.67 | -0.04 | -0.04 | -0.04 | -0.36 | -0.10 | -0.10 | -0.23 |  |
| USGlacier | 0 | 0 | 0 | 0 | 0 | 0.003 | 0.001 | 0 | 0 | 0 | 0 | 0 | 0 | 0.342 | 0.049 | 0 | 0.203 | 0 | 0 |  | 0.05 | 0.07 | -0.12 | -0.06 | 0.30 | 0.29 | 0.27 | 0.37 | 0.35 | 0.35 | -0.02 |  |
| ReachSed | 0 | 0 | 0 | 0 | 0 | 0 | 0 | 0 | 0 | 0 | 0 | 0 | 0 | 0 | 0 | 0 | 0 | 0 | 0 | 0 |  | 0.73 | -0.57 | -0.46 | -0.04 | -0.04 | -0.03 | 0.31 | 0.10 | 0.10 | 0.11 |  |
| ReachHab | 0 | 0 | 0 | 0 | 0 | 0 | 0 | 0 | 0 | 0 | 0 | 0 | 0 | 0 | 0 | 0 | 0 | 0 | 0 | 0 | 0 |  | -0.49 | -0.32 | -0.10 | -0.10 | -0.10 | 0.31 | 0.07 | 0.07 | 0.07 |  |
| DRP_State | 0 | 0 | 0 | 0.716 | 0 | 0 | 0 | 0 | 0 | 0 | 0 | 0 | 0 | 0 | 0 | 0 | 0 | 0 | 0 | 0 | 0 | 0 |  | 0.56 | -0.07 | -0.07 | -0.08 | -0.32 | -0.14 | -0.14 | -0.08 |  |
| NO3N_State | 0 | 0 | 0 | 0 | 0 | 0 | 0 | 0 | 0 | 0 | 0 | 0 | 0 | 0 | 0 | 0 | 0 | 0 | 0 | 0 | 0 | 0 | 0 |  | -0.04 | -0.04 | -0.04 | -0.21 | -0.08 | -0.08 | -0.14 |  |
| Q5 | 0.046 | 0 | 0 | 0 | 0 | 0 | 0 | 0.084 | 0 | 0 | 0 | 0.011 | 0.001 | 0.894 | 0.224 | 0 | 0.277 | 0 | 0 | 0 | 0 | 0 | 0 | 0 |  | 1.00 | 0.98 | 0.17 | 0.79 | 0.79 | -0.06 |  |
| MALF | 0.081 | 0 | 0 | 0 | 0 | 0 | 0 | 0.08 | 0 | 0 | 0 | 0.026 | 0.001 | 0.943 | 0.217 | 0 | 0.286 | 0 | 0 | 0 | 0 | 0 | 0 | 0 | 0 |  | 0.98 | 0.17 | 0.78 | 0.78 | -0.06 |  |
| MeanF | 0.351 | 0 | 0 | 0 | 0 | 0 | 0 | 0.062 | 0 | 0 | 0 | 0.112 | 0 | 0.634 | 0.139 | 0 | 0.252 | 0 | 0 | 0 | 0 | 0 | 0 | 0 | 0 | 0 |  | 0.16 | 0.78 | 0.78 | -0.04 |  |
| Feb | 0 | 0 | 0 | 0 | 0 | 0.003 | 0 | 0 | 0 | 0 | 0 | 0 | 0 | 0 | 0 | 0 | 0.959 | 0 | 0 | 0 | 0 | 0 | 0 | 0 | 0 | 0 | 0 |  | 0.32 | 0.32 | 0.13 |  |
| WidthQ5 | 0.031 | 0 | 0 | 0 | 0 | 0 | 0 | 0.125 | 0 | 0 | 0 | 0 | 0 | 0.028 | 0 | 0 | 0.001 | 0 | 0 | 0 | 0 | 0 | 0 | 0 | 0 | 0 | 0 | 0 |  | 1.00 | -0.08 |  |
| WidthMALF | 0.028 | 0 | 0 | 0 | 0 | 0 | 0 | 0.132 | 0 | 0 | 0 | 0 | 0 | 0.031 | 0 | 0 | 0.001 | 0 | 0 | 0 | 0 | 0 | 0 | 0 | 0 | 0 | 0 | 0 | 0 |  | -0.08 |  |
| FRE3 | 0 | 0 | 0 | 0 | 0 | 0 | 0 | 0 | 0 | 0 | 0 | 0 | 0 | 0 | 0 | 0.014 | 0 | 0 | 0 | 0.01 | 0 | 0 | 0 | 0 | 0 | 0 | 0 | 0 | 0 | 0 |  |  |
